# Robustness Can Evolve Gradually in Complex Regulatory Gene Networks with Varying Topology

**DOI:** 10.1371/journal.pcbi.0030015

**Published:** 2007-02-02

**Authors:** Stefano Ciliberti, Olivier C Martin, Andreas Wagner

**Affiliations:** 1 Laboratoire de Physique Théoique et Modèles Statistiques, Universite Paris-Sud, Orsay, France; 2 Centre National de la Recherche Scientifique, Universite Paris-Sud, Orsay, France; 3 Laboratoire de Genetique Vegetale du Moulon, Universite Paris-Sud, Gif-sur-Yvette, France; 4 L'Institut National de la Recherche Agronomique, Universite Paris-Sud, Gif-sur-Yvette; 5 Centre National de la Recherche Scientifique, Universite Paris-Sud, Gif-sur-Yvette, France; 6 Department of Biochemistry, University of Zurich, Switzerland; Harvard University, United States of America

## Abstract

The topology of cellular circuits (the who-interacts-with-whom) is key to understand their robustness to both mutations and noise. The reason is that many biochemical parameters driving circuit behavior vary extensively and are thus not fine-tuned. Existing work in this area asks to what extent the function of any one given circuit is robust. But is high robustness truly remarkable, or would it be expected for many circuits of similar topology? And how can high robustness come about through gradual Darwinian evolution that changes circuit topology gradually, one interaction at a time? We here ask these questions for a model of transcriptional regulation networks, in which we explore millions of different network topologies. Robustness to mutations and noise are correlated in these networks. They show a skewed distribution, with a very small number of networks being vastly more robust than the rest. All networks that attain a given gene expression state can be organized into a graph whose nodes are networks that differ in their topology. Remarkably, this graph is connected and can be easily traversed by gradual changes of network topologies. Thus, robustness is an evolvable property. This connectedness and evolvability of robust networks may be a general organizational principle of biological networks. In addition, it exists also for RNA and protein structures, and may thus be a general organizational principle of all biological systems.

## Author Summary

Living things are astonishingly complex, yet unlike houses of cards they are also highly robust. That is, they have persisted for billions of years, despite being exposed to an endless stream of environmental stressors and random mutations. Is this robustness an evolvable property? Do different biological systems vary in their robustness? Has natural selection shaped this robustness? These questions are very difficult to answer experimentally for most systems, be they proteins or large gene networks. Here we address these questions with a model of the transcription regulation networks that regulate both cellular functions and embryonic development in many organisms. We examine millions of such networks that differ in the topology or architecture of their regulatory interactions, that is, in the “who interacts with whom” of a network. We find that radically different network architectures can show the same gene expression pattern. The networks' robustness to both mutations and gene expression noise shows a broad distribution: some network architectures are highly robust, whereas others are quite fragile. Importantly, the entire space of network architectures can be traversed through small changes of individual regulatory interactions, without changing a network's gene expression pattern. This means that high robustness in gene expression can evolve through gradual and neutral evolution in the space of network architectures. Our results show that the robustness of transcriptional regulation networks is an evolvable trait that natural selection can change like any other trait.

## Introduction

The biochemical parameters that determine the behavior of cellular systems—from proteins to genome-scale regulatory networks—change continually. Such change has two principal sources. One of them is genetic and consists of mutations. The other is nongenetic; it is exemplified by noise internal to the organism and by environmental change. In contrast to mutations, which are relatively rare, internal noise is ubiquitous and substantial. Much of it consists of stochastic variation in gene expression and expression regulation [1–6]. Such noise makes all biochemical parameters affecting a circuit's behavior appear to fluctuate randomly. Environmental change, such as a change in temperature, salinity, or nutrient availability, can similarly affect many parameters at once. These observations suggest that biological circuits are not fine-tuned to exercise their functions only for precise values of their biochemical parameters. Instead, they must be able to function under a range of different parameters. In other words, they must be robust to parameter change. These insights have lead to explorations of circuit robustness in processes ranging from bacterial chemotaxis to embryonic development [7–16].

Quantitative models of cellular circuits help us to understand processes as different as circadian rhythms [17–25], the cell cycle [26], organismal development [7,9,10,16,27–31], bacterial chemotaxis [8], and the behavior of synthetic circuitry [32–36]. Several classes of models are used to represent such biological networks. The first class comprises differential equation models. The continuous state variables in these equations correspond to the concentrations or activities of gene products. The interactions of these gene products are represented through biochemical parameters such as binding affinities of transcriptional regulators to DNA, dissociation constants of ligand-receptor complexes, or kinetic rate constants of enzymes. A nearly universal problem is that quantitative information about these biochemical parameters is absent, even for experimentally well-studied systems. In other words, some knowledge of the topology of a circuit—who interacts with whom—may exist, but the strengths of the interactions are usually unknown. Even where measurements of biochemical parameters are available, they are often order-of-magnitude estimates rather than quantitative measurements with known precision. This difficulty leads one naturally to a second class of models in which only the qualitative nature of the state variables (on–off, or low–high) is considered.

Our focus here is not to consider any one circuit but many circuit architectures or topologies. Because of the incessant changes of biochemical parameters and the lack of quantitative information about their values, such an approach is appropriate for studying fundamental properties of cellular circuits; in particular, one may ask what features are responsible for the robustness of a circuit architecture or topology [7,9,29,37,38]. In this work, we carry out an analysis for a model of transcriptional regulation networks with important functions in developmental processes. Despite its level of abstraction, this model has proven highly successful in explaining the regulatory dynamics of early developmental genes in the fruit fly Drosophila as well as in predicting mutant phenotypes [27,39–41]. It has also helped to elucidate why mutants often show a release of genetic variation that is cryptic in the wild-type, and how adaptive evolution of robustness occurs in genetic networks of a given topology [42–45]. Most recently, it has helped explain how sexual reproduction can enhance robustness to recombination [46].

**Figure 1:**
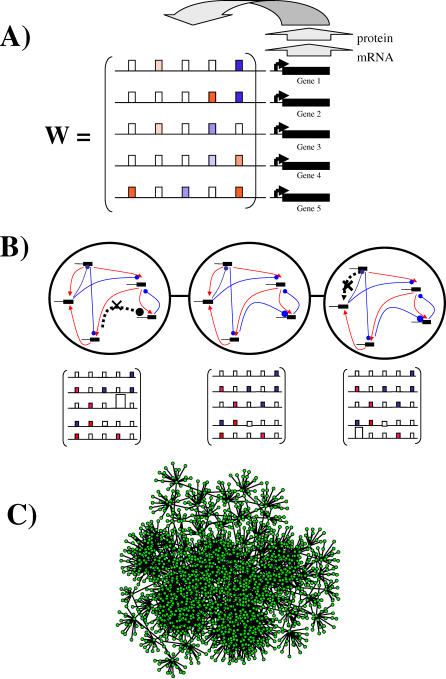
Regulatory Network Model and the Metagraph Concept (A) A transcriptional regulation network. Solid black bars indicate genes that encode transcriptional regulators in a hypothetical five-gene network or gene circuit. Each gene's expression state is influenced by the transcriptional regulators in the network. This influence is usually exerted by binding of a transcriptional regulator to a gene's regulatory region (horizontal line). The model represents the regulatory interactions between transcription factor *j* and genes *i* through a matrix *w* = (*w_ij_*). A regulator's effect can be activating (*w_ij_* > 0*,* red rectangles) or repressing (*w_ij_* < 0, blue rectangles). Any given gene's expression may be unaffected by most regulators in the network (*w_ij_* = 0, open rectangles). The different hues of red and blue correspond to different magnitudes of *w_ij_*. The highly regular correspondence of matrix entries to binding sites serves the purpose of illustration. We note that transcription factor binding sites often function regardless of their position in a regulatory region. (B) Gradual evolutionary changes and the metagraph. The middle panel shows a hypothetical network of five genes (top) and its matrix of regulatory interactions *w* (bottom), if genes are numbered clockwise from the uppermost gene. Red arrows indicate activating interactions and blue lines terminating in a circle indicate repressive interactions. The left-most network and the middle one differ in one repressive interaction from gene four to gene three (dashed gray line, black cross, large open rectangle). The right-most network and the middle one differ in one activating interaction from gene one to gene five (dashed line, black cross, large open rectangle). Each of the three network topologies corresponds to one node in a metagraph of network topologies, which is indicated by the large circle around the networks. These circles are connected because the respective networks are neighbors in the metagraph, i.e., they differ by one regulatory interaction. (C) Part of a metagraph for a regulatory network with *N* = 4 genes. Each node corresponds to a network of a given topology, and two nodes are connected by an edge if they differ at one regulatory interaction (*M ≈ 0.5 N^2^* regulatory interactions, and Hamming distance of **S**(0) and **S**_∞_ of *d* = 0.5). The metagraph in this case is connected, and the number of edges incident on a node is highly variable. The graph shown includes all viable networks that differ at no more than four regulatory interactions from an arbitrary node in the metagraph. Note that metagraphs typically have a huge number of nodes. The number of nodes in a metagraph can be counted because different nodes differ only in the signs of their regulatory interactions.

The model [42] is concerned with a regulatory network of *N* transcriptional regulators, which are represented by their expression patterns **S**(*t*) = (*S_1_*(*t*), *S_2_*(*t*), …, *S_N_*(*t*)) at some time *t* during a developmental or cell-biological process and in one cell or domain of an embryo. The time scale of the model's expression dynamics is the time scale characteristic for transcriptional regulation, which is on the order of minutes, and not on the order of days, weeks, or months, as for complete development from zygote to adult. The model's transcriptional regulators can influence each other's expression through cross-regulatory and autoregulatory interactions, which are encapsulated in a matrix *w* = (*w_ij_*). The elements *w_ij_* of this matrix indicate the strength of the regulatory influence that gene *j* has on gene *i* (Figure 1A). This influence can be either activating (w*_ij_* > 0), repressing (*w_ij_* < 0), or absent. Put differently, the matrix *w* represents the (regulatory) genotype of this system, while the expression state is its phenotype. We model the change in the expression state **S**(*t*) of the network (hereafter also referred to as a circuit) as time *t* progresses according to the difference equation 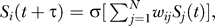 where *τ* is a constant, and *σ*(.) is a sigmoidal function whose values lie in the interval (−1, +1). This equation reflects the regulation of gene *i*'s expression by other genes. We are here concerned with networks whose expression dynamics start from a prespecified initial state **S**(0) at some time *t* = 0 during development, and arrive at a prespecified stable equilibrium or “target” expression state **S**_∞_. We will call such networks *viable* networks. The initial state is determined by regulatory factors upstream of the network, which may represent signals from the cell's environment or from other domains of an embryo. Transcriptional regulators that are expressed in the stable equilibrium state **S**_∞_ affect the expression of genes downstream of the network. As a modeling assumption, we think of their expression as critical for the course of development. Thus, deviations from **S**_∞_ are highly deleterious. It is because our work starts from such a developmental framework that **S**(0) and **S**_∞_ play a central role; this is in contrast with most studies determining the generic properties of random Boolean networks [30,31,37,38,47–50].

We here examine the relationship between robustness and network topology for millions of networks with different topologies. Topology is synonymous with the “structure” of the matrix *w,* because each of *w*'s nonzero entries corresponds to one regulatory interaction among the circuit's genes (Figure 1A). Changes in topology correspond to the loss of a regulatory interaction (*w_ij_* → 0), or to the appearance of a new regulatory interaction that was previously absent. Such topological changes can occur on very short evolutionary time scales, in particular in higher eukaryotes with large regulatory regions [51]. This underscores the need to study their effects on network robustness. In our analysis, we first ask how robustness to mutations and noise varies within an ensemble of networks with different topologies. Subsequently, and more importantly, we also ask whether highly robust topologies can evolve from topologies with low robustness through gradual topological changes.

## Results

### Robustness to Noise and Robustness to Mutations Are Highly Correlated

**Figure 2:**
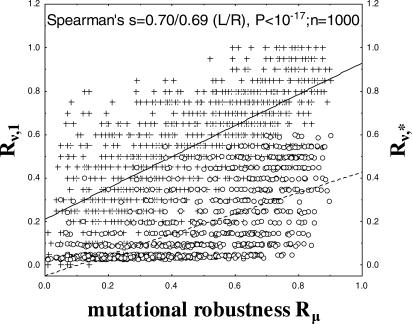
A High Statistical Association between Robustness to Mutations and to Noise The horizontal axis shows mutational robustness *R_μ_,* which is the fraction of a viable network's neighbors (networks differing from it in only one regulatory interaction) that arrive at the same equilibrium state **S**_∞_ given the initial state **S**(0). The vertical axes show two different measures of robustness to noise. The left vertical axis (+, solid line) shows *R_ν,_*_1_, the probability that a change in *one* gene's expression state in the initial expression pattern **S**(0) leaves the network's equilibrium expression pattern **S**_∞_ unchanged. The right vertical axis (circles, dashed line) shows R_ν,__*_, the fraction of genes whose expression state in **S**(0) has to change at random, such that the probability that a network arrives at the equilibrium state **S**_∞_ falls below a value of ½. In a network with large R_ν,__*_, perturbation of the expression states of a large fraction of genes affects the equilibrium pattern only rarely. *R_μ_* is highly associated with both *R_ν,_*_1_ (Spearman's *s* = 0.70) and R_ν,__*_ (Spearman's *s* = 0.69, *p* < 10^−15^; 10^3^ networks for both). The sample is obtained from a Monte Carlo simulation as described in Methods (*N* = 20, *M* ≈ 0.25 *N^2^* regulatory interactions, *d* = 0.5, *w_ij_* = ±1).

Robustness to mutations on one hand, and to environmental change and internal noise on the other hand, correspond to two different measures of robustness in the circuits we study. In both cases, the robust feature is the network's equilibrium gene expression pattern **S**_∞_. Robustness to mutations corresponds to robustness of **S**_∞_ to changes in regulatory interactions, either as a change in network topology, or as a change in the strength of regulatory interaction. Specifically, for a given *viable* network, we define mutational robustness *R_μ_* as the fraction of its one-mutant neighbors that are also viable. Robustness to noise corresponds to robustness of **S**_∞_ to changes in gene expression patterns. We use three complementary measures of robustness to noise. The first of them is the probability *R_ν,1_* that a change in *one* gene's expression state in the initial expression pattern **S**(0) leaves the network's equilibrium expression pattern **S**_∞_ unchanged. The second measure is the fraction R_ν__*_ of genes whose expression needs to change in **S**(0), such that the probability of attaining the equilibrium state falls below ½. The third measure is the probability that changes in the gene expression trajectory from **S**(0) to **S**_∞_ preserve **S**_∞_ (see Text S1 for details). Importantly, robustness to noise and robustness to mutations are highly correlated. Figures 2 and S1A illustrate this for one example, a network of *N* = 20 genes (Spearman's *s* > 0.56, *p* < 10^−15^). Similar observations have been made for mutational robustness and thermodynamic stability in RNA secondary structures [52].

We show in Text S1 that all important network properties depend only on the fraction of genes that differ in their expression state between **S**(0) and **S**_∞_. We refer to this fraction as the distance *d* between the two states (0 ≤ *d* ≤ 1). We find highly significant associations between our four measures of robustness for wide ranges of values for this distance *d,* the number of genes *N,* and number of regulatory interactions. Examples are shown in Tables S1 and S2 for two of our measures of robustness to noise. A comparison of the tables shows that the significant correlations between robustness to mutations and to noise exist regardless of how robustness to noise is assessed. The data in these tables are for networks where regulatory interactions are discrete (*w_ij_* = ±1), but the same conclusions hold for networks with continuous-valued regulatory interactions (Figure S1B; Tables S3 and S4).

### The Fraction of Viable Networks Is Tiny

Consider all networks of a given number *N* of genes and total number *M* of regulatory interactions. The fraction *v_f_* of viable networks, that is, networks that arrive at a prespecified target expression state **S**_∞_ given an initial gene expression state **S**(0), is generally tiny. We first present a qualitative argument for why this should be so. Consider an equilibrium expression state **S**_∞_. Now choose one network *w* at random out of the space of all possible networks. Because there are 2*^N^* possible equilibrium states, the probability that this network *w* arrives at **S**_∞_ should be at most on the order of 1/2*^N^*. This simple observation suggests that the fraction of viable networks should be exponentially small in *N*. A quantitative analysis for networks with *w_ij_* = ±1 confirms this exponential scaling (Figure S2). Even for small networks, the fraction *v_f_* of viable networks is small. For example, we used exhaustive enumeration to show that for networks with *N* = 4 genes (M = 8 regulatory interactions, *d* = 0.5) less than 0.5% of networks are viable. For moderately sized networks of *N* = 20 genes (*M* = 200, *d* = 0.5), random sampling establishes that *v_f_* = 5.1 × 10^−9^ ± 1.7 × 10^−10^. That is, fewer than one in one hundred million networks are viable.

### A “Metagraph” of Networks That Differ Greatly in Their Robustness

Next, we focus on the set of viable networks with a given number *N* of genes and a number *M* of regulatory interactions within a narrow range. From the set of these networks, we define a graph whose nodes correspond to the networks: two networks (nodes) in this graph are connected if they differ in the value of only one regulatory interaction (Figure 1B). We call this graph a *metagraph,* because it is a graph whose nodes are networks—which could themselves be represented as (oriented) graphs. These nodes differ in the topology of their regulatory interactions. Neighboring networks in the metagraph arise from one another by genetic changes that affect only one regulatory interaction (Figure 1B). In the biological evolution of network topology, this metagraph could be traversed through a series of small genetic changes, each affecting one regulatory interaction.

From here on we shall concentrate on mutational robustness. This is not much of a restriction since robustness to noise and to mutational robustness are highly correlated; thus, one can be used as a proxy for the other. Clearly, metagraphs are ideally suited to study how mutational robustness evolves. In fact, all questions about the evolution of mutationally robust regulatory network topologies are questions about the structure of the metagraph. We discuss most of our results for the case where regulatory interactions are discrete (*w_ij_* = ±1), but nearly all of our results hold also for regulatory interactions that have continuous values.

**Figure 3:**
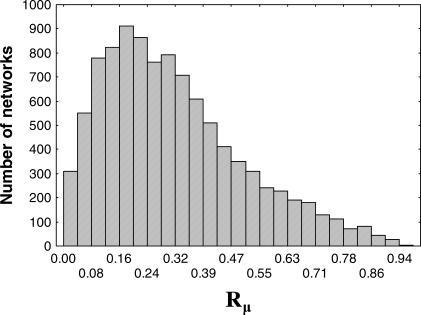
Heterogeneous Distribution of Mutational Robustness The histogram shows the distribution of the fraction *R_μ_* of neighbors of a network that differ at one regulatory interaction but attain the same equilibrium gene expression state **S**_∞_. The data is obtained from a sample of 10^4^ networks (*N* = 20; *M* ≈ 0.5 *N^2^; d* = 0.7) sampled from the metagraph in a Monte Carlo simulation as outlined in Methods.

The higher a network's mutational robustness *R_μ_* is, the larger the number of regulatory interactions one can change without affecting the network's equilibrium gene expression state **S**_∞_. If all nodes in the metagraph had the same number of neighbors, all networks would be equally robust, and robustness could not change in biological evolution. However, this is not the case. Figure 3 shows the distribution of mutational robustness for networks with *N =* 20 genes and *M* ≈ 0.25 *N^2^* regulatory interactions. There are clearly vast differences in robustness among networks. For example, the most robust network in Figure 3 has *R_μ_* = 0.98 and is almost 300-fold more robust than the network with the lowest robustness (*R_μ_* = 3.3 × 10^−3^). Figure S3 shows that qualitatively the same observations hold for networks with varying regulatory interactions and varying distance between **S**(0) and **S**_∞_. Networks with continuously valued regulatory interactions show a similarly broad distribution of robustness. All of these properties seem to be general, holding for mutational robustness and for our three measures of robustness (unpublished data). The distribution of robustness has no tendency to become more “concentrated” at a pronounced peak with increasing *N*. We thus cannot restrict ourselves to studying a “typical” *R_μ_*. In sum, different networks show very different robustness to mutations or noise, and heterogeneity in robustness is the rule.

### Networks Can Evolve Gradually toward Robustness

A key question regarding the evolvability of robust networks is whether one can reach highly robust networks starting from networks of low robustness through a series of small genetic changes. This is not self-evident. Recall that viable networks comprise a tiny fraction of all possible ones. They could be widely scattered in the space of all possible networks and occupy disconnected islands in this space. However, our analysis indicates precisely the opposite. The metagraph of viable networks has one “giant” connected component that comprises most or all viable networks. Any two networks in this component can be reached from one another through gradual changes of one regulatory interaction at a time, changes that never leave the space of viable networks.

We demonstrated these properties in the following manner. First, for networks with few genes, we can obtain all viable networks through exhaustive enumeration. In this case, we test whether the metagraph of viable networks is connected by comparing the whole list of viable networks to that associated with a connected component. This component is constructed by initiating a random walk on the metagraph, starting from an arbitrary viable starting network. The list of all *distinct* viable networks visited during this random walk is a lower bound on the size of the connected subpart of the metagraph that contains this starting network. This number usually is very close or equal to the total number of viable networks. For example, for networks of *N* = 5 genes, 6 ≤ *M* ≤ 7 regulatory interactions, and *d* = 0.4, there are a total of 37,338 viable networks (out of 6.3 × 10^7^ possible ones). A random walk visiting 10^7^ networks finds all 37,338 of these. More generally, long random walks through the space of viable networks visit all but a very small fraction of the nodes of the metagraph, and this missing fraction decreases as *N* increases.

Second, when *N* becomes too large to enumerate all viable networks, Monte Carlo sampling becomes necessary (see Text S1). For networks with few genes and few regulatory interactions (one to two interactions per gene), some randomly chosen viable networks are isolated points of the metagraph. We note that this situation is exceptional and results from our constraint that forbids more than a prespecified small total number of regulatory interactions. In the generic case, however, which becomes more and more prevalent as *N* increases, an overwhelming fraction of the whole metagraph is in one “giant component” (Table S5). For instance, a fraction 0.998 of viable networks belong to the giant component of the metagraph for *N* as small *N* = 12 (*M ≈* 0.25 *N^2^*, *d* = 0.5).

We conclude that most or all viable networks are contained in one large connected component for the cases we examined here. This means that nearly all viable networks can evolve toward greater robustness through gradual changes in topology. This great cohesiveness of viable networks is not self-evident, as we show in Text S1. Specifically, it does not hold for a metagraph comprising the same number *n_v_* of nodes as the above metagraph of viable networks, where neighboring nodes (networks) differ in one regulatory interaction, but where the nodes need not be viable. In such a “random metagraph,” the probability that an arbitrary node is isolated is bounded from below by [1 − *K*/(*n* − *n_v_* + 1)]^*n_v_*−1^ ≈ 1, where *K* is the number of neighbors of a network *w*. It follows immediately that only a negligible fraction of the nodes in a random metagraph is *not* isolated.

The connectivity difference between metagraphs of viable and of random networks is already drastic for a small number of genes. For example, for *N* = 6, *M* ≈ 0.5 *N^2^* regulatory interactions, and *d* = 0.5, there are a total of *n* = 8.59 × 10^13^ networks. Using random sampling, we find that there are *n_v_* = 7.77 × 10^6^ viable networks, of which only a fraction, 0.0022, is isolated. For a random metagraph of the same size, the fraction of isolated nodes would be at least 0.988.

For all of our previous analyses, we have considered only one pair of initial and target gene expression states. Regulatory gene networks, however, often function in more than one context inside the organism, each of which can be characterized by a different pair of initial and equilibrium states. A detailed analysis of such multiple gene expression states will be reported elsewhere. Here, we just note that our key results are unaffected by these additional constraints. Specifically, although for any given *N* and *M,* the metagraph of viable networks is more often disconnected than where there is a single initial-target pair, it is still dominated by a single connected giant component as *N* increases, and the networks in this component still show a broad distribution of robustness (Figure S5).

### Mutational Robustness and Natural Selection

All evolution by natural selection takes place in populations of organisms. To find out to what extent natural selection can change mutational robustness, one thus has to take into account the dynamics of an evolving population of organisms (networks) on the metagraph. Specifically, the question is to what extent natural selection can shape the average mutational robustness (or the robustness to noise) of networks in the population [53]. We here briefly summarize a relevant result from earlier work [54] that was derived with biological macromolecules in mind but applies also to the networks considered here. This result pertains to populations that evolve under the influence of (regulatory) mutations and strong selection to maintain viability. For small population sizes *P,* small number of genes *N,* or small mutation rates *μ* (*PNμ* ≪ 1), natural selection is not capable of increasing population robustness beyond the mean robustness of the networks in the metagraph. In contrast, for larger populations with sufficiently high mutation rates (*PNμ* ≫ 1), the population becomes concentrated at nodes (genotypes) of higher mutational robustness. To understand the selective force driving the evolution of high mutational robustness, consider two hypothetical subpopulations of networks on a metagraph, one with low robustness, the other with high robustness. Mutations arrive at the two subpopulations with equal frequency. However, individuals in the subpopulation with low robustness are much more likely to lose viability than individuals with high robustness. Over many generations, this preferential elimination of individuals with low robustness drives the evolution of high robustness. In the long run, the average robustness *R̄*_μ_ in a population of networks will exceed the mean of *R_μ_* when averaging uniformly over the metagraph. In fact, in the large population size limit, *R̄*_μ_ converges to an eigenvalue associated with the adjacency matrix [55] of the metagraph [54]. We here estimate *R̄*_μ_ numerically.

**Figure 4:**
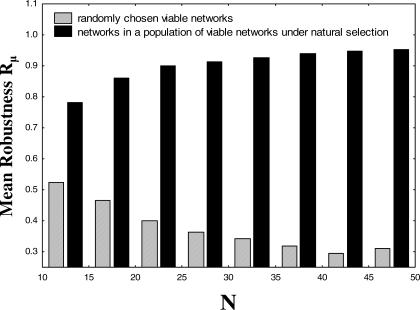
Natural Selection for Viability Dramatically Increases Robustness The horizontal axis shows the number of genes per network. Black bars on the vertical axis show the mean mutational robustness *R̄*_μ_ for populations under viability selection with *PNμ* ≫ 1, and grey bars show the mean mutational robustness *R̄*_μ_ for a population with *PNμ* ≪ 1, in which selection for increased robustness is ineffective, and, thus, sampling of the metagraph is uniform (*M* ≈ 0.25 *N^2^*, *d* = 0.5).

Figure 4 shows the mean population robustness *R̄*_μ_ for a large population subject to natural selection (black bars) and for a random sample of the metagraph (open bars) which represents the average robustness of networks in the metagraph. The difference is a measure of the extent to which natural selection can increase robustness. Figure 4 shows that although natural selection acts on viability alone, population robustness is enhanced compared with the metagraph average. Although this holds regardless of the number *N* of genes, the ratio of population robustness to average robustness increases with increasing *N,* rising to a factor of approximately three when *N* = 40. Larger values of *N* have greater potential to evolve increased robustness. The same holds for networks with continuously valued regulatory interactions (Figure S6) and for our different measures of robustness to noise (unpublished data).

### Designing Robust Networks

We here develop a “minimum-frustration” [56] prescription for the design of a highly robust network. There are two key requirements for robust network design. The first of them is that the equilibrium gene expression state should be highly stable, such that noise or mutations leave it unchanged. In this regard, we note that the expression *S_i_* of each gene in the equilibrium state has to fulfill the equation

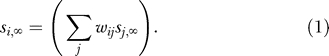



The equilibrium expression state will be most stable if the sum above is large in absolute value, because changes in individual gene expression states or regulatory interactions will not affect the sign of the sum. In the discrete case (*w_ij_* = ±1), the largest possible absolute value is achieved if one chooses *w_ij_* = *S_i_*_,*∞*_ *S_j_*_,*∞*_ for all nonzero regulatory interactions.

The second key requirement for a robust network is that the equilibrium gene expression state can be reached quickly from the initial state. The longer the network's trajectory to the equilibrium state, the greater the chance that the trajectory veers off course due to gene expression noise, and the smaller the network's mutational robustness. For example, in a sample of 10^4^ networks with *N* = 40 genes, *M* ≈ 100 regulatory interactions, and *d* = 0.5, *R_μ_* is highly correlated with the reciprocal of the time *t* needed to reach the equilibrium state (*R_μ_* − 1/t: *s* = 0.88, *p* <10^−15^).

In the discrete-time model we consider, the shortest possible time from initial to equilibrium state is one time step. Which networks have this shortest possible trajectory? To find out, it is best to separate the genes of the network into two groups, those that have the same (“aligned”) expression state in the initial and equilibrium expression pattern (*S_i_*(0) = *S_i_*_,*∞*_), and those that have a different (“misaligned”) expression state (*S_i_*(0) ≠ *S_i_*_,∞_). To reach the equilibrium state in just one step (the shortest possible amount of time), a network has to obey the equation

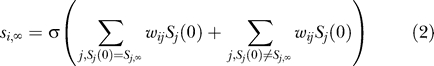



If one again chooses *w_ij_* = *S_i_*_,∞_ *S_j_*_,∞_ for all *j* belonging to “aligned” genes, then the left sum will make a contribution that is most favorable. In addition, this choice also favors the stability of the equilibrium state. For the group of “misaligned” genes, the opposite choice, e.g., *w_ij_* = −*S_i_*_,∞_ *S_j_*_,∞_ might seem appropriate, because it has the correct sign to validate the equation; however, this choice would directly oppose the stability of the equilibrium state.

Taken together, these observations suggest the following prescription for designing highly robust networks. For any gene *j* whose expression state is the same in the initial and the equilibrium states, choose *w_ij_* = *S_i_*_,∞_ *S_j_*_,∞_ whenever a regulatory interaction is present. For genes that are not of that type, we assign the magnitude of nonzero interactions *w_ij_* such that the right-hand sum in the above expression is zero or close to zero for every *i.* (For a sufficiently large total number of regulatory interactions, i.e., *M*/*N* » 1, choosing random values for these interactions will achieve this goal.)

We note that although the fraction of networks designed to be highly robust may be tiny, their absolute number may be astronomical for any given number of genes, initial states, and equilibrium states, simply because of the different ways to choose which regulatory interactions are present. (Furthermore, our prescription also leaves some freedom for choosing the strengths of those interactions that are to cancel out.) We also note that our prescription resembles the Hebb rule for storing information in neural networks [57], an important difference being that our networks are asymmetric (*w_ij_* ≠ *w_ji_*). In Text S1, we demonstrate that the simple principles discussed here are sufficient to produce highly robust networks (Figure S4).

## Discussion

To summarize, we find that networks of different topology vary by orders of magnitude in their robustness to mutations and noise. Highly robust networks can be “designed” following a simple prescription for their regulatory interactions. Most importantly, highly robust networks can be reached from networks with lower robustness through gradual evolutionary change, one regulatory interaction at a time. Not only that, all or most networks with a given equilibrium gene expression state are connected in one large metagraph of network topologies. These findings hold for a wide range of numbers of genes, total numbers of regulatory interactions, and different initial and equilibrium gene expression patterns.

Albeit the subject of some earlier work [50,58], the topology of regulatory networks has received more widespread attention since the realization that many biological systems keep functioning when faced with a wide spectrum of genetic and nongenetic change. Such change alters the biochemical parameters—concentrations, binding constants, half-lives, etc.—under which a network operates. It *requires* studying network topologies if one is to understand robustness, because much else about a network is in constant flux. Important earlier work has largely focused on the extent to which one or few network or circuit topologies supported by experimental data are robust [7,9,10]. The assertion that such networks are indeed robust has a major limitation: how do we know that their robustness is unusual or remarkable? This question can only be answered by studying many network topologies and their *distribution* of robustness. The same holds for our central question: how can robustness can be achieved through gradual Darwinian evolution, a process that does not create radical new network architectures in one step, but slowly modifies existing networks? An evolutionary perspective becomes important here: although circuits with a desirable feature may exist, it may be impossible to reach them through gradual evolution from other circuits. The difficulty in answering these questions is due to insufficient *empirical* information on topological variants of any one specific biological network.

What is the value of our results, given that they are based on a general model of transcriptional regulation networks, and not on one specific network in one organism? Results from such an abstract model have the advantage that they may apply to all or most networks that share specific characteristics. In this regard, we note that our model is designed to capture the qualitative behavior of transcriptional regulation networks in which cooperative regulation of gene expression is important. Given how central such cooperative regulation is in eukaryotes, it is perhaps not surprising that variants of this model can correctly predict the gene expression dynamics of biological circuits such as the Drosophila gene regulation network [27,39–41]. Also, our key findings do not depend strongly on many details such as the number of genes or regulatory interactions. Finally, similar results—broad distribution and evolvability of robustness—have been recently reported for a small sample of circadian oscillator networks that are very different from our regulatory model [59], which suggests that robustness may be evolvable for a broad class of cellular networks. At the least, our results call for analysis of a wide range of experimentally well-understood circuits with partially known topology, in order to find out whether there are biologically important exceptions to our findings.

The evolution of increased robustness by the mechanism discussed here is neutral evolution. This does not mean that all mutations that occur in the process are neutral. Some mutations in regulatory interactions—those that cause a network to leave the metagraph—may be deleterious. However, such mutations are eliminated by natural selection, and only the neutral mutations survive. If we had sufficient data to study the evolution of transcriptional regulation networks over long times, for example by following changes in transcription factor binding sites, then the deleterious mutations that inevitably occur during evolution would leave only one trace: conservation—to a greater or lesser extent—of individual binding sites. Limited conservation of regulatory interactions and binding sites [60,61] is thus no contradiction to neutral evolution. It just indicates that some mutations that occurred in the evolutionary history of a network have been eliminated by natural selection.

We note intriguing parallels of our observations to the work of others both on artificial systems, such as “digital organisms,” and on natural systems, such as the sequence–structure relationships of RNA and protein molecules [52,62–70]. The secondary or tertiary structure of a molecule can be viewed as its phenotype (analogous to the gene expression pattern of a regulatory network). Its RNA or amino acid sequence is its genotype (analogous to a regulatory network with a given topology of regulatory interactions). The set of all molecules (sequences) that adopt the same structure comprises both sequences of great and little robustness to mutations or thermal noise. Most importantly, a set of sequences adopting the same structure typically form a very large connected graph called a neutral network, where sequences differing only at one residue are neighbors in the graph; our notion of a metagraph for regulatory circuits mirrors the neutral network concept. Such topological properties show that gradual evolution changing one residue at a time—analogous to changes affecting one regulatory interaction at a time—can readily traverse such a graph and find highly robust sequences, or sequences that have any other desirable feature. The observation that robustness is evolvable in biological systems at two different levels of organization—molecule and circuit—with different architectures and purposes, further suggests that our findings reflect a general organizational principle of biological systems.

## Methods

### Random sampling of viable networks.

To explore the space of random viable networks, we generate such networks numerically with uniform probability when this space is discrete (for instance, when the regulatory interaction strengths are either zero or ±1). A random network is easily generated by assigning to each of the *N^2^* values *w_ij_* a random value. However, in our study we also constrain the number *M* of nonzero *w_ij_* to lie in a given range, (*M*_−_,*M*_+_). To meet this constraint, we first compute the fraction of networks that have each of the allowed values of *M*. This allows us to generate a probability distribution for *M* within the allowed range. For any one randomly chosen *M,* we then choose at random the *M* nonzero interactions. This procedure uniformly samples the space of all networks, satisfying the range constraint on *M*. Keeping only those networks that are viable then leads to a uniform sampling of the space of viable networks, allowing us to estimate parameters of interest, such as the distribution of robustness. This algorithm can be extended to continuous interaction strengths (see Text S1).

### Exploring the connectivity properties of the space of viable networks.

To show that the space of viable networks is indeed dominated by one large connected component, we first start with some arbitrary viable reference network. Then we determine numerically the fraction of viable networks that can be connected to this reference network via some series of point changes in the interaction weights. To do this, we generate a random viable network; from it, we produce a long sequence of 10^6^ point changes that are randomized but preferentially reduce the Hamming distance to the reference network. If during this sequence we reach the reference network, then the two networks are manifestly “connected”; otherwise, we consider that the two of them are not connected to one another. We repeat this procedure for 1,000 random reference viable networks. In practice, we find that nearly all (more than 99%) of the networks are “connected” to the reference one.

## Supplementary Information

Figure S1Statistical Association between Robustness to Mutations and to Noise(A) The horizontal axis shows robustness to perturbations of transient expression changes, *R*_trans_. *R*_trans_ is the probability that a network still reaches **S**_∞_, as estimated from 5*N* perturbed trajectories of the dynamical system 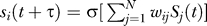 , where, during each time step, we pick one gene *i* at random, and reset its expression value randomly. The vertical axes show mutational robustness *R_μ_* and *R_ν,1_* as defined in the text. *R_trans_* is highly associated with both *R_μ_* (Spearman's *s* = 0.57) and *R_ν,1_* (Spearman's *s* = 0.76, *p* < 10^−15^; 10^3^ networks for both). R_ν,__*_ is also highly correlated with *R_trans_* (Spearman's *s* = 0.56, *p* < 10^−17^), but the values are not plotted here for visual clarity. The sample is obtained from a Monte Carlo simulation as described in Methods (*N* = 20, *M* ≈ 0.25 *N^2^* regulatory interactions, *d* = 0.5, *w_ij_* = ±1).
(B) Analogous to Figure 2, except for continuous regulatory interactions. The horizontal axis shows mutational robustness *R_μ_,* which is the fraction of a network's neighbors (networks differing in only one regulatory interaction) that arrive at the same equilibrium state S_∞_ given the initial state S(0). The vertical axes show two different measures of robustness to noise. The left vertical axis (+, solid line) shows *R_ν,1_,* the probability that a change in one gene's expression state in the initial expression pattern S(0) leaves the network's equilibrium expression pattern S_∞_ unchanged. The right vertical axis (circles, dashed line) shows R_ν__*_, the fraction of genes whose expression state in S(0) has to change at random, such that the probability that a network arrives at the equilibrium state **S**_∞_ falls below ½. In a network with large R_ν__*_, perturbation of the expression states of a large fraction of network genes affects the equilibrium pattern only rarely. *R_μ_* is highly associated with both *R_ν,1_* (Spearman's *s* = 55) and R_ν__*_ (Spearman's *s* = 0.48, *p* < 10^−15^; 10^3^ networks for both). The sample is obtained from a Monte Carlo simulation as described in Methods (*N* = 20, *M* = ≈0.25 *N^2^*, *d* = 0.5).(343 KB PPT)

Figure S2 The Fraction of Viable Networks Is Tiny The horizontal axis shows the number of genes (*N*). The vertical axis shows the fraction of viable networks *v_f_* for discrete regulatory interactions *w_ij_* = ±1) on a logarithmic scale. The number of interactions is *M* ≈ 0.5 *N^2^,* and the distance *d* between initial and equilibrium state is set to *d* = 0.5. Note the exponential decrease of *v_f_* with increasing *N*. The middle line (▵) indicates numerical estimates of *v_f_* obtained through random sampling from the space of all possible networks. Sampling errors are at least one order of magnitude smaller than the estimated values and thus invisible on the plot. The upper and lower lines indicate analytically obtained upper and lower bounds on *v_f_*, respectively. The upper bound is the fraction of networks that have **S**_∞_ as their equilibrium state. The lower bound corresponds to the fraction of viable networks that reach **S**_∞_ from S(0) in the shortest possible time (*t* = 1). (These bounds can be computed without sampling and are thus exact.)
(45 KB PPT)

Figure S3 Heterogeneous Distribution of Mutational Robustness for Different Values of *c* (*M ≈ cN^2^*) and *d*
All four histograms show the distribution of the fraction *R_μ_* of neighbors of a network that differ at one regulatory interaction but attain the same equilibrium gene expression state **S**_∞_. While the shape of this distribution may depend on network parameters, two important features vary little: the most robust networks represent a very small fraction of networks, and the difference in robustness between the most and least robust networks can be very large. The data shown are based on a sample of 10^4^ networks (*N* = 20) sampled from the metagraph in Monte Carlo simulation as outlined in Methods. The total number of regulatory interactions is *cN*(*N − 1*) *≤ M ≤ cN^2^*. Values of *c* and *d* are indicated in (A–D).
(76 KB PPT)

Figure S4 Association between Mutational Robustness and Quality Q of a Network The scatterplot shows the values of *R_μ_* and the quantity *Q* defined in the main text for a sample of 10^4^ viable networks with *N* = 40 genes, *M* ≈ 0.5 *N^2^,* and *d* = 0.5. Networks with high values of *Q* also have high robustness *R_μ_* (Spearman's *s* = 0.65, *p* < 10^−15^, 10^4^ networks).
(166 KB PPT)

Figure S5 A Broad Distribution of Robustness for More Than One Initial-Target Pair of Gene Expression States The figure shows a histogram of values of *R_μ_* based on 1,300 randomly sampled networks with *N* = 12 genes, *M* ≈ 0.25 *N^2^* regulatory interactions, and two pairs of input-target gene expression states with randomly generated initial and equilibrium gene expression states.
(43 KB PPT)

Figure S6 Natural Selection for Viability Dramatically Increases Robustness The horizontal axis shows the number of genes per network. Results are based on populations that are under viability selection. Black bars on the vertical axis show the mean mutational robustness *R̄*_μ_ for populations with *PNμ* ≫ 1, where selection is effective, and grey bars show the mean mutational robustness *R̄*_μ_ for populations with *PNμ* ≪ 1, in which selection is ineffective, and where, thus, sampling of the metagraph is uniform (*M* ≈ 0.5 *N^2^, d* = 0.5, continuous-valued regulatory interactions *w_ij_*).
(46 KB PPT)

Table S1 Robustness to Mutations and to Noise *R_ν,__1_* Are Associated Regardless of the Number *N* of Genes, Fraction *c* of Regulatory Interactions ( *M* ∼ *cN^2^* ), and the Distance *d* between Initial and Equilibrium State Shown are Spearman correlation coefficients (*p*-values) between *R_μ_* and *R_ν,1_* for *n* = 1,000 networks randomly sampled as described in Methods. Regulatory interactions are discrete (*w_ij_* = ±1). NS, nonsignificant.
(20 KB DOC)

Table S2 Robustness to Mutations and to Noise R_ν,__*_ Are Associated Regardless of the Number *N* of Genes, Fraction *c* of Regulatory Interactions (*M* ≈ *cN^2^*), and the Distance *d* between Initial and Equilibrium State Shown are Spearman correlation coefficients (*p*-values) between *R_μ_* and R_ν,*_ for *n* = 1,000 networks randomly sampled as described in Methods. Regulatory interactions are discrete (*w_ij_* = ±1). NS, nonsignificant.
(20 KB DOC)

Table S3 Robustness to Mutations and to Noise *R_ν,1_* Are Associated Regardless of the Number *N* of Genes, Fraction *c* of Regulatory Interactions (*M* ∼ *cN^2^*), and the Distance *d* between Initial and Equilibrium State Shown are Spearman correlation coefficients (*p*-values) between *R_μ_* and *R_ν,1_* for *n* = 1,000 networks randomly sampled as described in Methods. Regulatory interactions are continuous. NS, nonsignificant.
(22 KB DOC)

Table S4 Robustness to Mutations and to Noise R_ν,__*_ Are Associated Regardless of the Number *N* of Genes, Fraction *c* of Regulatory Interactions (*M* ≈ *cN^2^*), and the Distance *d* between Initial and Equilibrium State Shown are Spearman correlation coefficients (*p*-values) between *R_μ_* and R_ν,__*_ = 1,000 networks randomly sampled as described in Methods. Regulatory interactions are continuous. NS, nonsignificant.
(22 KB DOC)

Table S5 Few Networks Are Not in the Giant Connected Metagraph Component, and Most of These Networks Are Isolated Values in columns 3 and 4 were obtained as outlined in the Text S1 section “Numerically determining metagraph connectivity for large networks” for samples of *V* = 10^5^ (*N* = 6,8) and *V* = 10^4^ (*N* = 12, 20) randomly chosen viable networks. Values preceded by < might be equal to zero, but this cannot be ascertained numerically. Total number of regulatory interactions *M* ∼ *cN^2^*.
(55 KB DOC)

Text S1 Supplementary Material (204 KB DOC)
