# Supplementary material for: Robustness Can Evolve Gradually in Complex Regulatory Gene Networks with Varying Topology
Source: PLoS Comput Biol. 2007 Feb 2;3(2):e15. doi: 10.1371/journal.pcbi.0030015 (PMC1794322; doi:10.1371/journal.pcbi.0030015)
Supplement: Supplementary file 1 [file pcbi.0030015.sd001.doc]

Text S1. Supplementary Material

**Motivation**

Modeling complex regulatory networks can rarely be done analytically. It is thus important to develop numerical tools for analyzing such networks. One major difficulty is the huge dimension (or more simply the volume) of the space to explore, even when the number of genes is quite modest.: Generally it will only be possible to *sample* this space, but even that can be a non trivial task. A second difficulty arises if one is not interested in typical properties (for instance properties that arise in more than 0.1% of randomly chosen networks): even if only a small fraction, say of 1 in a million networks have a desired property, it is important to be able to construct these networks and to understand their essential features. Here we show how a number of computational approaches can be usefully applied to overcome these obstacles. Although we developed these approaches in the context of our model of regulatory networks, they should prove to be of more general interest.

**The model**

**Gene expression dynamics**

In our model genetic networks, the equation giving the change with time of the expression state is

$S_{i}(t+\tau)=\sigma[\sum w_{\text{ij}}S_{j}(t)]$	(1)

A more detailed biological motivation for this equation has been presented elsewhere [42]. It is analogous to equations used in neural computation [57,71]. We use synchronous updating of the state variables *S_i_*. This can affect the detailed phenotype of a given genotype, but it will not affect the states that are left invariant by the iterations. Furthermore, if a circuit is robust, using asynchronous rather than synchronous dynamics is unlikely to change its phenotype. (See [26,30] for case studies where the different choices of the dynamics are shown to lead to minor changes.) The function σ(.) should be monotonic and without loss of generality can be taken to map its argument to the interval (-1,1). In the limit of a very steep slope at the origin, σ(.) becomes the step function giving (-1) for negative arguments and (+1) for positive ones; this is the limit we consider for all the work described in this article. (Furthermore, we also set σ(0)=0.) However, the steep sigmoidal function used in others studies of these networks [43] would yield results identical to those reported here.

Given an initial gene expression state **S**(0) (at some time defined as *t*=0 during development), a network may attain at a later time an equilibrium expression state **S**_∞_. Since our space of expression states is finite in size, any equilibrium state has to be reached in at most 2*^N^* time steps. However, to examine that many time steps numerically is prohibitive; fortunately we find that if an equilibrium state is reached, it is found in far fewer steps. More specifically, in our study of small and intermediate *N*, all equilibrium states are reached after fewer than *N^2^* time steps. Thus our working definition of a *viable* network is that it attains the equilibrium state **S**_∞_ from **S**(0) in fewer than *N^2^* time steps. Note that networks with extremely long transients of more than *N^2^* time steps would have marginal biological relevance anyway, because their dynamics are exceedingly fragile to perturbations. (As we discuss in the section “designing robust networks”, there is a strong negative association between the length of a transient and a network’s robustness.)

**Mutational Dynamics**

We here outline how a network genotype (specified by *w*) is allowed to change through mutations. The two constraints on such mutational change are that only one regulatory interaction can be modified at a time, and that the number *M* of regulatory interactions must stay in a given range ($M_{-}$,$M_{+}$), for reasons given in the section “Generating random walks on the metagraph”. We call two networks (viable or not) that fulfill the second constraint *nearest neighbors* if they differ by just one regulatory interaction.

For any one network, many possible mutations may fulfill the above constraints. We take all the allowed mutations to be equiprobable in the case of discrete regulatory interactions ($w_{\text{ij}}=\pm1$). The case of continuous regulatory interactions is more subtle, because it implies an infinite number of possible mutations. Instead of a probability for each mutational change, we must introduce a *probability density*. We do this by distinguishing three different cases. A mutation may cause (i) an existing interaction to disappear, in which case the respective *w_ij_* is set to zero, (ii) a new regulatory interaction to appear, in which case the new value is chosen as a Gaussian random variable with mean zero and variance one (N(0,1)), and (iii) an existing interaction to change in magnitude. This last case has no counterpart for the discrete model. For such a mutation, we force the sign of the interaction to remain constant by choosing an N(0,1) random variable and multiplying it by (-1) if it is of the wrong sign. Each putative interaction *w_ij_* to be mutated is chosen at random; if the interaction is already present, we take the mutations of cases (ii) and (iii) to be equiprobable. Of course, as in the discrete case, we only consider those mutations keeping the total number of interactions in the range ($M_{-}$,$M_{+}$).

Lastly, we must define what we mean by a “random” network; this means introducing a *measure* (in the mathematical sense) over all networks. In the case of discrete regulatory interactions ($w_{\text{ij}}=\pm1$), we use the uniform measure, where all networks are equiprobable. In the case of continuous regulatory interactions, we first introduce a probability *p(M)* for the number *M* of non-zero interactions; this probability is the same as for the discrete case, namely it is given by the number of ways of assigning *M* non-zero interactions with their signs (but ignoring their magnitude) among *N^2^* possible interactions, divided by the number of such ways, and summed over all *M* values in the range ($M_{-}$,$M_{+}$). Then for a given value of *M*, the probability density of a network is obtained by considering each non-zero interaction value to be a Gaussian (N(0,1)) random variable.

**Supplementary Methods**

**Unbiased sampling of the set** ***W* of networks with a given initial and equilibrium state, and measures of robustness**

We here outline how to find the fraction of networks that are viable, given a number *N* of genes, a pair of initial and equilibrium states ($S(0)$,$S_{\infty}$), and a range for the number *M* of regulatory interactions. This is not a trivial problem, because the set of viable networks $W=\left\{ w \right\}$ such that $S(0)\vec{w}S_{\infty}$ is astronomical in size and thus precludes an exhaustive enumeration except for rather small *N*. What we need is a procedure for sampling the set *W* *uniformly*. The simplest procedure is to generate an interaction matrix at random; this can be done relatively efficiently for small *N* while respecting the constraints on *M*. However, this procedure becomes impractical for large *N*, because the fraction of viable networks then becomes very small. We thus also implemented a modified procedure which uses the gauge symmetry discussed later: if a randomly generated network *w* takes the initial state **S**(0) to an equilibrium state $S^{'_{\infty}}$ with the same distance as that between $S_{\infty}$ and **S**(0), then *w* can be mapped to a *w*’ in *W* by a permutation of indices and a gauge transformation. Such matrices *w*’ can then be used to measure the size of *W*. We applied this approach also to our analysis of multiple initial-equilibrium state pairs (thereby significantly reducing the statistical error compared to that of direct random sampling).

For each of the networks generated by this approach, we determine its mutational robustness *R_μ_* (its fraction of viable neighbors), as well as three different measures of robustness to noise. The first of them, *R_ν,1_* is the probability that a change in a single gene’s expression state in **S**(0) preserves the equilibrium state$S_{\infty}$. The second measure, *R_ν*_*, is determined as follows. We change, at random, a fraction $s\in(0,1)$ of the gene expression states in the initial state $S(0)$, and estimate the probability *q(s)* that the network arrives at the equilibrium state $S_{\infty}$, given a perturbation of magnitude *s*. *q(s)* monotonically decreases from 1 (*s*=0) to 0 (*s*=1), and we estimate the value of *s* at which *q(s)* falls below ½. The third measure, *R_υ,trans_*, is the probability that changes in the gene expression trajectory from **S**(0) to$S_{\infty}$ preserve $S_{\infty}$. We estimate *R_υ,trans_* from 5*N* perturbed trajectories of the dynamical system (1), where, during each time step, we pick one gene *i* at random, and reset its expression value randomly.

**Generating random walks on the metagraph**

In the main text we examined the connectivity properties of the metagraph by using random walks on its nodes, stepping from one network to a neighboring one, all the time restricting ourselves to viable networks. We generated these random walks - leading to a sampling of that part of the metagraph connected to the starting node - with the Metropolis Monte Carlo algorithm at zero temperature [72]. After motivating constraints on the total number *M* of regulatory interactions, we discuss details of the algorithm for discrete regulatory interactions ($w_{\text{ij}}=\pm1$). However, its key elements are identical for continuous *w_ij_*’s.

Throughout all of our work, the number *M* of regulatory interactions is forced to stay approximately constant, i.e., within a narrow range ($M_{-}$,$M_{+}$). Note that constraining the number of regulatory interactions to one value would preclude any evolutionary change. We use mainly the constraint *cN(N*-1)≤*M*≤*cN^2^*, where 0≤*c*≤0.5 is a constant. Broadening or narrowing this interval has no qualitative effect on our results (data not shown). The restriction to *c*≤0.5 is motivated biologically by the generally sparse connectivity of biological networks. For example, the network involved in the patterning of the syncytial *Drosophila* embryo comprises 15 genes with 32 interactions among them [73]. Among 76 transcriptional regulators in the yeast *Saccharomyces cerevisiae*, 106 putative regulatory interactions exist in the form of transcription factors bound to the promoter regions of other transcription factor coding genes [74,75]. Among 55 transcriptional regulators of *E. coli*, 61 transcriptional regulation interactions exist [76]. Note that allowing for at least two consecutive values of the number of regulatory interactions is necessary if one wants to explore the effect of regulatory mutations that eliminate or add regulatory interactions one at a time.

The Monte Carlo algorithm needs a starting point, which in our case is a network *w*. The simplest possibility is that of a *random start*, that is, a random network with *N* genes and *M* regulatory interactions. The problem is that with large *N* and *M*, the probability that such a network *w* is in the set *W* can be very small and the network *w* can thus be very difficult to find. We thus use an alternative strategy, which we refer to as “*cold start*”. Here, the starting point is a network *w* that, following our prescription for designing highly robust networks, has a high probability to be viable. Starting with such a network, the algorithm involves changing the network *w* step-by-step, one regulatory interaction at a time, creating a chain of networks *w,* *w^’^,…* in *W*. At each step, we first pick at random a regulatory interaction $w_{\text{ij}}$ of the current network, say *w*. If$w_{\text{ij}}=0$, its value is set to (+1) or (-1) with respective probabilities ½; if $w_{\text{ij}}\neq0$, its value is set to zero. However, if either of these rules would take the number *M* of regulatory interactions outside the bounds ($M_{-}$,$M_{+}$), a new$w_{\text{ij}}$ is chosen at random, until one is found whose change does not violate these bounds; simply put, one chooses at random one of the neighbors of the current network. Finally, we test whether the resulting network *w’* arrives at the equilibrium state ($S(0)\vec{w'}S_{\infty}$); if it does, one accepts the change according to the Metropolis rule [72], so *w*’ becomes the new current network. Otherwise, *w*’ is rejected and the current network is again *w*. (The same strategy applies to continuous-valued$w_{\text{ij}}$’s, where we use Gaussian-valued regulatory interactions as described above.) This whole process defines one step of the Monte Carlo simulation, a step that is repeated many times. This approach has the desirable property that for a sufficiently large number of steps, the connected component is sampled *uniformly*.

**Numerically determining metagraph connectivity for large networks**

Our procedure to determine metagraph connectivity has two phases. In the first phase, we generate a large connected component *C_c_* of the metagraph by (1) generating a network *w_0_* designed to be viable; (2) performing a long (>10^6^ steps) random walk on the metagraph that starts from this network. We record all *distinct* networks visited during this random walk, and call this set *C_c_*. In the second phase, we determine the probability with which a random viable network of the metagraph lies in the same connected component as the networks in *C_c_*. This is done as follows. First, we choose a viable network *w* at random, and construct a path through the space of viable networks by successively changing individual regulatory interactions. At each step in this path, we determine whether the current network belongs to *C_c_*. If so, we know that *w* can be connected to *C_c_* and hence that *w* belongs into the same metagraph component as *C_c_*. If after at least 10^5^ time steps the walk has not “encountered” *C_c_*, we consider that *w* is *not* connected to *C_c_*. The mean number *V* of randomly chosen viable networks that can be connected to *C_c_* in this manner provides a lower bound for the fraction of the overall metagraph that belongs to one large connected component. We note that the metagraph is so large that the lower bound becomes poor (equal to zero) for moderately large *N*. To remedy this problem, we guide the random walks so that they are biased towards *w_0_*, which renders the lower bound close to one even for large *N*.

**Supplementary Results**

**Simple properties that are useful to characterize the space of networks**

In our system where each gene *i* can only be ‘on’ or ‘off’ ($S_{i}=\pm1$), there are 2*^N^* possible initial states and 2*^N^* possible equilibrium states for networks with *N* genes. Thus, the number of possible input-equilibrium state pairs is an astronomical 2^2^*^N^*, suggesting that it is hopeless to characterize the space of viable networks reliably. However, a few simple, qualitative considerations show otherwise.

The first of these considerations is based on a so-called gauge transformation

[77]. Consider a pair of input and equilibrium states, and a network *w* whose dynamics leads to the equilibrium state $S_{\infty}$ given the initial state $S(0)$, i.e., $S(0)\vec{w}S_{\infty}$. Now take an arbitrary index *k* which labels one of the *N* genes. The gauge transformation for our system corresponds to changing the sign of $S_{k}(t)$and of any element of *w* associated with that index. More precisely, if $i,j\neq k$ , then $w_{\text{ij}}$ is left invariant while $w_{\text{ik}}$ and $w_{\text{kj}}$both change sign. (In matrix language, the *k*-th row and columns of *w* are multiplied by (-1), and thus the diagonal term $w_{\text{kk}}$is left unchanged.) The resulting matrix *w’* corresponds to a network that may no longer arrive at $S_{\infty}$ given $S(0)$. However, it is easy to see that if one changes $S(0)$to $S'(0)$ by changing the sign of$S_{k}(0)$ (associated with the *k*-th gene), and if one changes $S_{\infty}$to $S'_{\infty}$by changing the sign of $S_{k_{,\infty}}$, then the new network arrives at $S'_{\infty}$given $S'(0)$. We can thus write $S'(0)\vec{w'}S'_{\infty}$. It is important to note that the change of *w* to *w’* is a one-to-one transformation that can be readily inverted.

A second useful symmetry of this dynamical system ($S(0)\vec{w}S_{\infty}$) corresponds to a swapping of two arbitrary indices *k_1_* and *k_2_*. For a matrix *w*, this operation induces a swap of the corresponding rows as well as the corresponding columns, and produces a matrix *w’*. If one generates a new initial state $S'(0)$by swapping the value of $S_{k_{1}}(0)$with that of $S_{k_{2}}(0)$, and a new equilibrium state $S'_{\infty}$by swapping the value of $S_{k}$with that of $S_{k_{2},\infty}$, one has again $S'(0)\vec{w'}S'_{\infty}$. This procedure can be repeated at will: the property holds not only for the swapping of any two indices, but for arbitrary permutations of any number of indices. Put differently, the dynamical system represented by *w* transforms “simply” when one reorders the genes. Again, any such permutation or reordering is one-to-one and can thus be inverted.

Both the gauge symmetry and the transformation under permutations can be used to reduce the complexity of our computational tasks considerably. Consider two arbitrary pairs of input and equilibrium states, and a corresponding network *w* ($S(0)\vec{w}S_{\infty}$). Next define the following transformation *T* of *w*. For each *k* such that the equilibrium gene expression state is $S_{k_{,\infty}}=-1$, change the sign of the corresponding row and then column of *w*. After this transformation, we have a new network $S'(0)\vec{w'}S'_{\infty}$ for which all entries of the equilibrium state $S'_{\infty}$are equal to (+1). Next define a permutation of the columns of *w* that corresponds to a reordering of the indices for the genes, so that in the initial state all expression levels equal to (+1) come before those equal to (-1). That is, the new initial state has the structure $(+1,+1,+1,\text{.}\text{.}\text{.},-1,-1,-1)$. Taken together, the gauge transformation and the permutation constitute a transformation *T(w)* that converts the original dynamical system, represented by *w*, into a new dynamical system *T(w)* whose equilibrium state (derived from $S_{\infty}$) contains only values (+1), and whose initial state (derived from $S(0)$) contains as many values of (-1) as the number of genes whose expression states differ in $S(0)$and$S_{\infty}$.

Because *T(w)* is a one-to-one transformation, all relevant properties of *w* map onto those of *T*(*w)*. More generally, consider the set $W=\left\{ w \right\}$ of all dynamical systems for which $S(0)\vec{w}S_{\infty}$holds for the original, arbitrary, initial and equilibrium states. The number of its elements |*W*| is exactly equal to the number of matrices |*T(W)|* that adopt the new equilibrium state, given the new initial state. In addition, the structure of the metagraph of all networks (matrices *w*) for which $S(0)\vec{w}S_{\infty}$is invariant under the transformation *T*. In other words, the structure of the metagraph for *any* pair of (arbitrary) initial and equilibrium states that differ at *p* out of *N* gene expression values is isomorphic (identical up to relabeling of genes and changes of signs of expression levels) to that of the metagraph for an equilibrium state $S_{\infty}$ containing only values (+1) and for an initial state $S(0)$that contains exactly *p* values equal to (-1). In sum, we have reduced the analysis of 2^2^*^N^* dynamical systems (all pairs of initial and equilibrium states) to an analysis of just (*N*+1) dynamical systems, differentiated by the number *p* of values equal to (-1) in the initial state. We distinguish these (*N+1)* dynamical system by *d*=*p*/*N*, that is, by the Hamming distance between initial and equilibrium state normalized to (0,1).

# Designing robust networks

To assess whether our design rule for robust networks really produces robust networks, we define the quantity *Q* (for network “quality”) as

$$Q=\frac{\sum\sum w_{\text{ij}}(S_{i,\infty}S_{j,\infty})}{\sum|w_{\text{ij}}|}$$

*Q* ranges from zero to a maximum value of one, which is attained by some networks that follow our prescription. If our prescription is correct, then networks with high Q should also have high robustness, and *Q* should be statistically associated with robustness. Note that there may be many networks with high robustness that may have sub-maximal values of *Q*, because we have only provided a sufficient criterion for robustness. Figure S4 shows the relation between *Q* and mutational robustness *R_μ_* in a sample of 10^4^ networks with *N*=40 genes, *M*≈0.25*N^2^* non-zero interactions, and *d*=0.5. Networks close to the maximum value of *Q* have the highest possible *R_μ_*. Numerical analysis shows that the two quantities are also significantly correlated (Spearman’s *s*=0.65, *P*<10^-15^, 10^4^ networks). In addition, as we would expect, both *Q* and *R_μ_* are correlated with the reciprocal of the time *t* needed to reach the equilibrium state (e.g., *Q*-1/*t*: *s*=0.69; *R_μ_*-1/*t*: *s*=0.88, *P*<10^-15^ for both). Similarly high associations are observed for robustness to noise (e.g., *R_ν,1_*-*Q*: s= 0.62; *R_ν,1_*-1/*t*: *s*=0.73; *P*<10^-15^). In sum, networks that follow our prescription are indeed highly robust.

# Estimation of the mean robustness attainable through natural selection

For large populations and populations with high mutation rates, selection for mutational robustness will generally be strongest, and the robustness attainable through evolution by natural selection for such robustness will attain its highest possible value. Specifically, the average population robustness $\bar{R}_{\mu}$of such a population of networks evolving under the influence of mutation and strong selection approaches the largest eigenvalue λ_max_ [54] of the matrix having for its entries *a_ij_* = 1/*K_i_* where *K_i_* is the total number of neighbors of network *i*. In our case, this matrix is generally not symmetric (*a_ij_* ≠ *a_ji_*). Up to the factor *K_i_^-1^*, this matrix is identical to the adjacency matrix [55] of the metagraph. The metagraph of even modestly sized networks is typically too large for one to calculate this eigenvalue directly using algebraic methods. However, we can use this important result to estimate $\bar{R}_{\mu}$ through a population evolution process, which proceeds as follows.

We begin with a finite but large population of *n(0)* identical viable networks (*n(0)*= 10^3^-10^5^). In each generation, each individual undergoes a single mutation to a neighboring network. Any individual that has mutated to a nonviable network is eliminated from the population. This cycle of mutation and selection is reiterated with the remaining individuals. If *n(0)* is very large, the number of individuals left after *T* generations would asymptotically decay as *n(T)* ~ const × λ_max_*^T^*. The parameter λ_max_ in this decay processes can be estimated from$\text{ln}\lambda=\lim_{T\to\infty}\left( \text{ln}{\frac{n(T)}{n(0)}}/T \right)$. For this estimation, *T* should be large enough so that the transient (non-exponential) behavior of the first iterations becomes negligible. Unfortunately, this is infeasible in practice because for sufficiently large *T* the population size will have decayed to zero. However, this problem can be circumvented by periodically doubling the population size (making one copy of each individual) to keep it in the range [*n*(0)/2, *n*(0)].

The theoretical result λ_max_=$\bar{R}_{\mu}$ [54] holds in the limit of an infinite population size. In practice we find that these two quantities – namely the decay rate λ_max_ of the population (correcting for the times its size has been doubled) on the one hand, and the mean mutational robustness of its individuals on the other – differ by O(1/*n*(0)) and that *n*(0)=10^4^ is sufficient for their relative difference to be smaller than 1% . We note that these population simulations are not designed to be biologically realistic. Their intention is to measure $\bar{R}_{\mu}$ precisely. Also, we emphasize that our results hold for strong viability selection only. Although it has been suggested that the evolutionary dynamics of robustness might be little changed for weaker selection [54], we do not explore this scenario here.

Everything said thus far pertains to mutational robustness. However, noise is a much more frequent perturbation than mutations, and the resulting selection pressure can be strong even in small populations. If even moderate sensitivity to noise is deleterious or lethal, a population in selection-mutation equilibrium may contain only the most highly robust networks. Such networks will show increased robustness to mutations as a correlated response (Figures 2 and S1). For example, for a sample of 10^4^ networks with *N*=20 genes (*c*=0.25, *d*=0.5), the mean mutational robustness *R_μ_* of networks with the maximal robustness to noise *R_ν,1_*=1 is *R_μ_*=0.78±0.006, 77% higher than the metagraph average of *R_μ_*=0.44.

The extent to which selection can increase robustness in large populations (acting on both mutational robustness and robustness to noise through viability) and in small populations (acting on robustness to noise through viability) may be very similar. For example, for networks with *N*=20 genes (*c*=0.25, *d*=0.5), natural selection can increase *R_μ_* from a metagraph average of *R_μ_*=0.44 to a population average of *R_μ_*=0.88, i.e., by a factor of two (Figure 4). The most highly robust networks in the metagraph have *R_μ_*=0.98. The metagraph average for robustness to noise of the same networks is *R_ν,1_*=0.55, whereas the most highly robust networks have *R_ν,1_*=1, a factor 1.8 difference. Thus, natural selection for robustness to noise may increase robustness to a higher value (*R_ν,1_*=1 vs. *R_μ_*=0.98), but not necessarily to a greater extent than natural selection for mutational robustness (a factor 1.8 versus a factor 2 increase for our example here).

**Random metagraphs consist mostly of isolated nodes**

A random metagraph comprises the same number *n_v_* of networks as a metagraph of viable networks, but its nodes (networks) need not be viable. (Neighboring networks in the random metagraph differ by one regulatory interaction.) Let *n* be the total number of networks for a given number of genes and regulatory interactions. Consider an arbitrary node *w* of the random metagraph. We want to calculate the probability that this node is isolated in the random metagraph, which will be the case if all the remaining *n_v_* -1 nodes in the random metagraph are distinct from the *K* nodes that comprise *w*’s neighbors. First, we determine the probability that a network *w’* chosen at random from the *n_v_* -1 remaining networks is different from these *K* networks. This probability is (1 – *K*/(*n* -1)). The probability that a second network *w*’’, chosen at random from the *n_v_* -2 remaining networks, is also different from these *K* networks is (1 – *K*/(*n* -2)). The argument proceeds analogously for a third, fourth, etc. network, until one arrives at the probability (1 – *K*/(*n* - *n_v_*+1)) that a last, (*n_v_* -1)^th^ network, is different from these *K* networks. The probability of interest is the product of all these probabilities. Note that each probability is smaller than the preceding one, so that their product is greater than

$$\left( 1-\frac{K}{n-n_{v}+1} \right)^{n_{v}-1}\approx1-\frac{(n_{v}-1)K}{n-n_{v}+1}\approx1$$

The left approximation holds, because *K* is of order *N^2^*, whereas the denominator is dominated by *n*, which scales exponentially in *N*. In addition, (*n_v_*-1)*K* / (*n* - *n_v_*+1)<<1, because we showed earlier that *n_v_* was exponentially small compared to *n,* while *K* is no greater than *N^2^*. Thus, the product (*n_v_*-1) *K* divided by *n* is exponentially small.

In sum, the probability that an arbitrary network *w* in the random metagraph is isolated is very close to one. It immediately follows that the average number of components of the random metagraph, given by *n_v_* times the above probability, is just slightly smaller than the total number of elements: only a negligible fraction of the nodes of the random metagraph are *not* isolated.

**Additional Literature Cited in Text S1**

71. Hertz J, Krogh A, Palmer RG (1991) Introduction to the theory of neural computation. Redwood City (California): Addison, Wesley. 352 p.

72. Metropolis N, Rosenbluth A, Rosenbluth M, Teller A, Teller E (1953) Equations of state calculations by fast computing machines. J Chem Phys 21: 1087–1092.

73. Carroll SB, Grenier JK, Weatherbee SD (2001) From DNA to diversity. Molecular genetics and the evolution of animal design. Malden (Massachusetts): Blackwell. 192 p.

74. Lee T, Rinaldi N, Robert F, Odom D, Bar-Joseph Z, et al. (2002) Transcriptional regulatory networks in *Saccharomyces cerevisiae*. Science 298: 799–804.

75. Evangelisti A, Wagner A (2004) Molecular evolution in the transcriptional regulation network of yeast. J Exp Zool/Mol Dev Evol 302B: 392–411.

76. Thieffry D, Huerta A, Perez-Rueda E, Collado-Vides J (1998) From specific gene regulation to genomic networks: A global analysis of transcriptional regulation in *E. coli*. BioEssays 20: 433–440.

77. Toulouse G (1977) Theory of the frustration effect in spin glasses: I. Commun Phys 2: 115–119.

**Supplementary Figure Captions**

**Figure S1. Statistical association between robustness to mutations and to noise.**

**A)** The horizontal axis shows robustness to perturbations of transient expression changes, *R*_trans._ *R*_trans_ is the probability that a network still reaches $S_{\infty}$, as estimated from 5*N* perturbed trajectories of the dynamical system $S_{i}(t+\tau)=\sigma[\sum w_{\text{ij}}S_{j}(t)]$ , where, during each time step, we pick one gene *i* at random, and reset its expression value randomly. The vertical axes show mutational robustness *R_μ_*, and *R_ν,1_*, as defined in the text. *R_trans_* is highly associated with both *R_μ_* (Spearman’s *s*=0.57) and *R_ν,1_* (Spearman’s *s*=0.76, *P*<10^-15^; 10^3^ networks for both). *R_ν,*_* is also highly correlated with *R_trans_* (Spearman’s s=0.56, P<10^-17^), but the values are not plotted here for visual clarity. The sample is obtained from a Monte Carlo simulation as described in Methods (*N*=20, *M*≈0.25*N*^2^ regulatory interactions, *d*=0.5, $w_{\text{ij}}=\pm1$).

**B)** Analogous to Figure 2, except for continuous regulatory interactions. The horizontal axis shows mutational robustness *R_μ_*, which is the fraction of a network’s neighbors (networks differing in only one regulatory interaction) that arrive at the same equilibrium state **S**_∞_ given the initial state **S**(0). The vertical axes show two different measures of robustness to noise. The left vertical axis (‘+’, solid line) shows *R_ν,1_*, the probability that a change in one gene’s expression state in the initial expression pattern **S**(0) leaves the network’s equilibrium expression pattern **S**_∞_ unchanged. The right vertical axis (circles, dashed line) shows *R_ν*_,* the fraction of genes whose expression state in **S**(0) has to change at random, such that the probability that a network arrives at the equilibrium state $S_{\infty}$falls below 1/2. In a network with large *R_ν*_* , perturbation of the expression states of a large fraction of network genes affects the equilibrium pattern only rarely. *R_μ_* is highly associated with both *R_ν,1_* (Spearman’s *s*=55) and *R_ν*_* (Spearman’s *s*=0.48, *P*<10^-15^; 10^3^ networks for both). The sample is obtained from a Monte Carlo simulation as described in Methods (*N*=20, *M*=≈0.25*N^2^*, *d*=0.5).

**Figure S2:** **The fraction of viable networks is tiny.** The horizontal axis shows the number of genes (*N*). The vertical axis shows the fraction of viable networks *v_f_* for discrete regulatory interactions ($w_{\text{ij}}=\pm1$) on a logarithmic scale. The number of interactions is *M*≈0.5*N*^2^, and the distance *d* between initial and equilibrium state is set to *d*=0.5. Note the exponential decrease of *v_f_* with increasing *N*. The middle line (▲) indicates numerical estimates of *v_f_* obtained through random sampling from the space of all possible networks. Sampling errors are at least one order of magnitude smaller than the estimated values and thus invisible on the plot. The upper and lower lines indicate analytically obtained upper and lower bounds on *v_f_*, respectively. The upper bound is the fraction of networks that have $S_{\infty}$as their equilibrium state. The lower bound corresponds to the fraction of viable networks that reach $S_{\infty}$ from **S**(0) in the shortest possible time (*t*=1). (These bounds can be computed without sampling and are thus exact.)

Figure S3. Heterogeneous distribution of mutational robustness for different values of *c* (*M≈cN^2^* ) and *d*. All four histograms show the distribution of the fraction *R_μ_* of neighbors of a network that differ at one regulatory interaction but attain the same equilibrium gene expression state$S_{\infty}$. While the shape of this distribution may depend on network parameters, two important features vary little: The most robust networks represent a very small fraction of networks, and the difference in robustness between the most and least robust networks can be very large. The data shown are based on a sample of 10^4^ networks (*N*=20) sampled from the metagraph in Monte Carlo simulation as outlined in Methods. The total number of regulatory interactions is *cN(N-1)≤M≤cN^2^*. Values of *c* and *d* are indicated in panels A)-D).

**Figure S4. Association between mutational robustness and quality Q of a network.** The scatter plot shows the values of *R_μ_* and the quantity *Q* defined in the main text for a sample of 10^4^ viable networks with *N*=40 genes, *M*≈0.5*N^2^*, and *d*=0.5. Networks with high values of *Q* also have high robustness *R_μ_*_._(Spearman’s *s*=0.65, *P*<10^-15^, 10^4^ networks).

**Figure S5.** A broad distribution of robustness for more than one initial-target pair of gene expression states. The figure shows a histogram of values of *R_μ_* based on 1300 randomly sampled networks with *N*=12 genes, *M*≈0.25*N^2^* regulatory interactions, and two pairs of input-target gene expression states with randomly generated initial and equilibrium gene expression states.

Figure S6. Natural selection for viability dramatically increases robustness.

The horizontal axis shows the number of genes per network. Results are based on populations that are under viability selection. Black bars on the vertical axis show the mean mutational robustness $\bar{R}_{\mu}$ for populations with *PNμ*>>1, where selection is effective, and grey bars show the mean mutational robustness $\bar{R}_{\mu}$ for populations with *PNμ*<<1, in which selection is ineffective, and where thus sampling of the metagraph is uniform (*M*≈0.5*N*^2^, *d*=0.5, continuous-valued regulatory interactions *w_ij_*).
