# Supplementary material for: Robustness Can Evolve Gradually in Complex Regulatory Gene Networks with Varying Topology
Source: PLoS Comput Biol. 2007 Feb 2;3(2):e15. doi: 10.1371/journal.pcbi.0030015 (PMC1794322; doi:10.1371/journal.pcbi.0030015)
Supplement: Supplementary file 3 [file pcbi.0030015.st002.doc]

**Table S2: Robustness to mutations and to noise are associated regardless of the**

**number *N* of genes, fraction *c* of regulatory interactions (*M*≈*cN*^2^) and the distance *d* between initial and equilibrium state.** Shown are Spearman correlation coefficients (*P*-values) between *R_μ_* and *R_ν,*_* for *n*=1000 networks randomly sampled as described in methods. Regulatory interactions are discrete ($w_{ij}=\pm1$). “NS” means non-significant.

c=0.1	d=0	d=0.2	d=0.5	d=0.7

N=12	NS	NS	NS	NS

N=20	NS	0.30 (<10^-15^)	0.49 (<10^-15^)	0.56 (<10^-15^)

N=40	0.17 (<10^-5^)	0.68 (<10^-15^)	0.80 (<10^-15^)	0.71 (<10^-15^)

c=0.25	d=0	d=0.2	d=0.5	d=0.7

N=12	0.10 (0.002)	0.43 (<10^-15^)	0.52 (<10^-15^)	0.56 (<10^-15^)

N=20	0.19 (<10^-5^)	0.59 (<10^-15^)	0.70 (<10^-15^)	0.56 (<10^-15^)

N=40	0.25 (<10^-15^)	0.82 (<10^-15^)	0.81 (<10^-15^)	0.75 (<10^-15^)

c=0.5	d=0	d=0.2	d=0.5	d=0.7

N=12	0.23 (<10^-10^)	0.46 (<10^-15^)	0.57 (<10^-15^)	0.56 (<10^-15^)

N=20	0.25 (<10^-15^)	0.63 (<10^-15^)	0.59 (<10^-15^)	0.40 (<10^-15^)

N=40	0.29 (<10^-15^)	0.81 (<10^-15^)	0.82 (<10^-15^)	0.78 (<10^-15^)
