# Supplementary material for: Robustness Can Evolve Gradually in Complex Regulatory Gene Networks with Varying Topology
Source: PLoS Comput Biol. 2007 Feb 2;3(2):e15. doi: 10.1371/journal.pcbi.0030015 (PMC1794322; doi:10.1371/journal.pcbi.0030015)
Supplement: Supplementary file 4 [file pcbi.0030015.st003.doc]

**Table S3: Robustness to mutations and to noise are associated regardless of the**

**number *N* of genes, fraction *c* of regulatory interactions (*M*=*cN*^2^) and the distance *d* between initial and equilibrium state.** Shown are Spearman correlation coefficients (*P*-values) between *R_μ_* and *R_ν,1_* for *n*=1000 networks randomly sampled as described in methods. Regulatory interactions are continuous. “NS” means non-significant.

c=0.1	d=0	d=0.2	d=0.5	d=0.7

N=12	NS	NS	NS	NS

N=20	0.07 (0.017)	NS	0.17 (<10^-5^)	0.27 (<10^-15^)

N=40	0.28 (<10^-15^)	0.33 (<10^-20^)	0.64 (<10^-15^)	0.68 (<10^-15^)

c=0.25	d=0	d=0.2	d=0.5	d=0.7

N=12	0.23 (<10^-10^)	0.06 (0.046)	0.36 (<10^-15^)	0.37 (<10^-15^)

N=20	0.32 (<10^-15^)	0.26 (<10^-15^)	0.55 (<10^-15^)	0.54 (<10^-15^)

N=40	0.42 (<10^-15^)	0.45 (<10^-15^)	0.36 (<10^-15^)	0.28 (<10^-15^)

c=0.5	d=0	d=0.2	d=0.5	d=0.7

N=12	0.34 (<10^-15^)	0.18 (<10^-5^)	NS	0.42 (<10^-15^)

N=20	0.46 (<10^-15^)	0.36 (<10^-15^)	0.24 (<10^-15^)	0.13 (<10^-4^)

N=40	0.50 (<10^-15^)	0.50 (<10^-15^)	0.50 (<10^-15^)	0.49 (<10^-15^)
