# Supplementary material for: Robustness Can Evolve Gradually in Complex Regulatory Gene Networks with Varying Topology
Source: PLoS Comput Biol. 2007 Feb 2;3(2):e15. doi: 10.1371/journal.pcbi.0030015 (PMC1794322; doi:10.1371/journal.pcbi.0030015)
Supplement: Supplementary file 6 [file pcbi.0030015.st005.doc]

**Table S5: Few networks are not in the giant connected metagraph component, and**

**most of these networks are isolated.** Values in columns 3 and 4 were obtained as outlined in “Numerically determining metagraph connectivity” for samples of *V*=10^5^ (*N*=6,8) and *V*=10^4^ (*N*=12, 20) randomly chosen viable networks. Values preceeded by “<” might be equal to zero, but this cannot be ascertained numerically. Total number of regulatory interactions *M*≈*cN*^2^.

| N | c | d | Fraction of networks not in giant component (upper bound) | Fraction of isolated networks |
| --- | --- | --- | --- | --- |
| 20 | 0.25 | 0.5 | 1.4×10^-3^ | 1.4×10^-3^ |
| 20 | 0.5 | 0.5 | 1.4×10^-3^ | 1.4×10^-3^ |
| 12 | 0.25 | 0 | <10^-4^ | <10^-4^ |
| 12 | 0.25 | 0.25 | 1.0×10^-4^ | 1.0×10^-4^ |
| 12 | 0.25 | 0.5 | 1.7×10^-3^ | 1.7×10^-3^ |
| 12 | 0.25 | 0.75 | 3.1×10^-3^ | 3.1×10^-3^ |
| 12 | 0.5 | 0 | <10^-4^ | <10^-4^ |
| 12 | 0.5 | 0.25 | 7.0×10^-4^ | 7.0×10^-4^ |
| 12 | 0.5 | 0.5 | 1.2×10^-3^ | 1.2×10^-3^ |
| 12 | 0.5 | 0.75 | 1.8×10^-3^ | 1.8×10^-3^ |
| 8 | 0.25 | 0 | <10^-5^ | <10^-5^ |
| 8 | 0.25 | 0.25 | 3.6×10^-4^ | 3.6×10^-4^ |
| 8 | 0.25 | 0.5 | 2.3×10^-3^ | 2.3×10^-3^ |
| 8 | 0.25 | 0.75 | 7.5×10^-3^ | 7.5×10^-3^ |
| 8 | 0.5 | 0 | <10^-5^ | <10^-5^ |
| 8 | 0.5 | 0.25 | 7.4×10^-4^ | 7.4×10^-4^ |
| 8 | 0.5 | 0.5 | 1.3×10^-3^ | 1.3×10^-3^ |
| 8 | 0.5 | 0.75 | 4.2×10^-3^ | 4.2×10^-3^ |
| 6 | 0.25 | 0 | <10^-5^ | <10^-5^ |
| 6 | 0.25 | 0.25 | 7.5×10^-4^ | 7.5×10^-4^ |
| 6 | 0.25 | 0.5 | 1.3×10^-2^ | 1.3×10^-2^ |
| 6 | 0.25 | 0.75 | 2.5×10^-2^ | 2.5×10^-2^ |
| 6 | 0.5 | 0 | <10^-5^ | <10^-5^ |
| 6 | 0.5 | 0.25 | 4.8×10^-4^ | 4.6×10^-4^ |
| 6 | 0.5 | 0.5 | 3.0×10^-3^ | 2.9×10^-3^ |
| 6 | 0.5 | 0.75 | 5.8×10^-3^ | 5.3×10^-3^ |
